# The Application of an Ultrasound Tomography Algorithm in a Novel Ring 3D Ultrasound Imaging System

**DOI:** 10.3390/s18051332

**Published:** 2018-04-25

**Authors:** Chang Liu, Chenyang Xue, Binzhen Zhang, Guojun Zhang, Changde He

**Affiliations:** 1 Key Laboratory of Instrumentation Science & Dynamic Measurement, North University of China, Ministry of Education, Taiyuan 030051, China; liuchang870820@126.com (C.L.); zhangguojun1977@nuc.edu.cn (G.Z.); hechangde@nuc.edu.cn (C.H.); 2 Science and Technology on Electronic Test and Measurement Laboratory, North University of China, Taiyuan 030051, China; 3 School of Electrical and Electronic Engineering, Dalian Vocational Technical College, Dalian 116037, China

**Keywords:** 3D imaging ring system, PMUT array, circular scanning, ultrasound tomography

## Abstract

Currently, breast cancer is one of the most common cancers in women all over the world. A novel 3D breast ultrasound imaging ring system using the linear array transducer is proposed to decrease costs, reduce processing difficulties, and improve patient comfort as compared to modern day breast screening systems. The 1 × 128 Piezoelectric Micromachined Ultrasonic Transducer (PMUT) linear array is placed 90 degrees cross-vertically. The transducer surrounds the mammary gland, which allows for non-contact detection. Once the experimental platform is built, the breast model is placed through the electric rotary table opening and into a water tank that is at a constant temperature of 32 °C. The electric rotary table performs a 360° scan either automatically or mechanically. Pulse echo signals are captured through a circular scanning method at discrete angles. Subsequently, an ultrasonic tomography algorithm is designed, and a horizontal slice imaging is realized. The experimental results indicate that the preliminary detection of mass is realized by using this ring system. Circular scanning imaging is obtained by using a rotatable linear array instead of a cylindrical array, which allows the size and location of the mass to be recognized. The resolution of breast imaging is improved through the adjustment of the angle interval (>0.05°) and multiple slices are gained through different transducer array elements (1 × 128). These results validate the feasibility of the system design as well as the algorithm, and encourage us to implement our concept with a clinical study in the future.

## 1. Introduction

Breast cancer is the most common cancer in women worldwide. To decrease the mortality rate of breast cancer, detection in the early stages with screening is crucial [1,2]. Mammography, hand-held ultrasound, computerized tomography (CT) and magnetic resonance imaging (MRI) are commonly used methods for breast-cancer screening [3]. However, many of these methods expose the examinee to harmful radiation and compression pain. Thus, an alternative screening system is necessary. A promising modality for imaging of breast cancer is ultrasound computer tomography (USCT) [4,5]. The benefits of USCT have been known for a long time and the first publications date back to the 70 s [6]. The USCT system is radiation-free, painless, and suitable for all breast categories [7]. One of the main advantages of the USCT system is the simultaneous recording of reflection, attenuation, and speed of sound images [8,9]. Additionally, high image quality and fast data acquisition can be achieved [10]. The patient is imaged in the prone position with the breast hanging freely into the imaging volume [11] so that the USCT images of the female breast are produced. Until now, building such a device has been extremely costly, and the processing technology of the big ring ultrasonic transducer array has been complex [12,13].

Breast cancer has higher density and sound speed, likely due to changes in cancerous tissue’s mechanical and elastic properties [14]. Mean values from the published sound reports are as follows: fat, 1478 m/s; glandular breast, 1510 m/s; benign breast tumors, 1513 m/s; and malignant breast tumors, 1548 m/s, suggesting that sound speed can be used to assess breast density and potentially detect breast cancer [15,16]. Research on breast ultrasound sound speed tomography goes back more than 35 years with the first report on transmission ultrasound tomography being published in 1974 [17]. In 1981, results were reported of a transmission mode breast scanner that simultaneously imaged pulse-echo backscatter, attenuation, and speed, which showed clear delineation of breast architecture [18,19]. More recent research has continued to demonstrate advancement in breast ultrasound tomography with most approaches using both transmission and reflection methods [20,21,22,23,24]. Currently, the B-Mode ultrasonic tomography is used broadly in medical ultrasonic imaging [25,26,27,28].

In this project, a 3D ultrasound imaging ring system using piezoelectric micro-machined ultrasonic transducer (PMUT) linear array is developed. The 1 × 128 PMUT linear array utilizes the ring system operating at a center frequency of 3.5 MHz. The ultrasonic transmit/receive signals are controlled through 16 channels acquisition circuit. C-Mode ultrasonic tomography is combined with PMUT to perform imaging of the breast model. Various experimental projects are used to validate feasibility of the system and validity of the algorithm.

## 2. 3D Ultrasound Imaging Ring System Description

**Figure 1:**
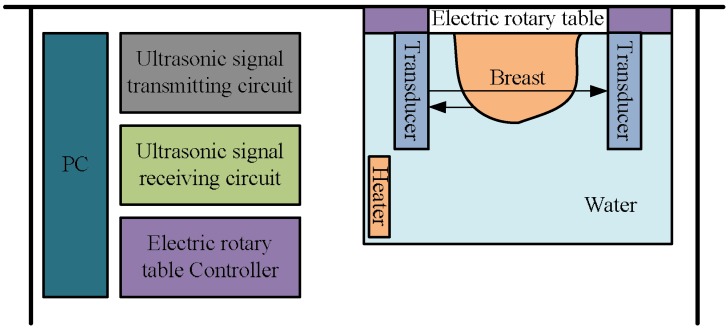
Diagram of the 3D ultrasound imaging ring system.

A novel 3D ultrasound imaging ring system is provided in this paper and the diagram is shown in Figure 1. It consists of the personal computer workstation, four linear ultrasonic transducer linear arrays, an electric rotary table and its controller, the storage water tank, ultrasonic signal transmitting and receiving circuits, and a constant temperature heater.

**Figure 2:**
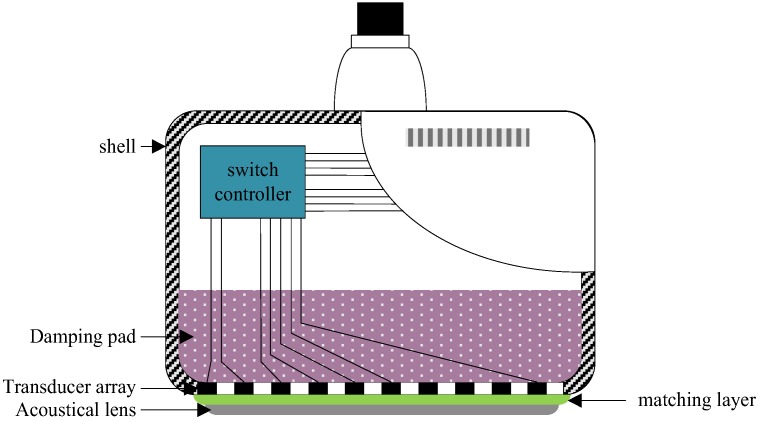
The cross-sectional structure of the 1 × 128 PMUT array.

**Figure 3:**
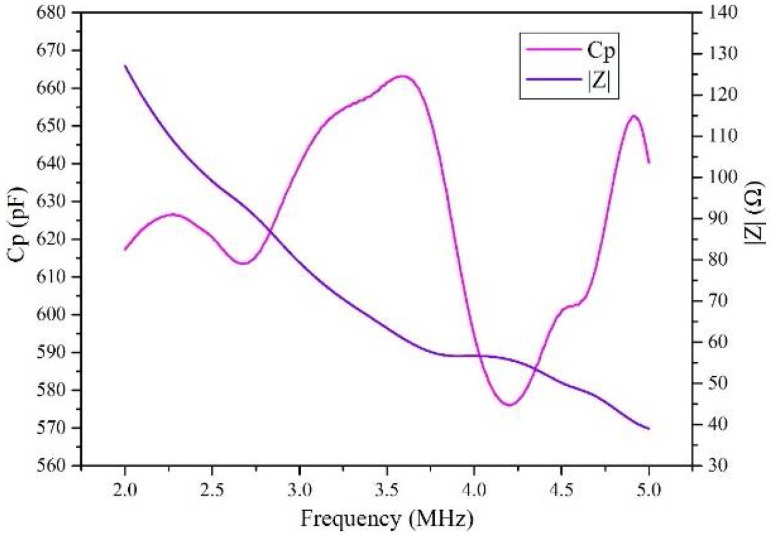
Frequency property of the PMUT.

The main components are described below:

(a) Linear ultrasonic transducer array:

The cross-sectional structure of the 1 × 128 PMUT array is shown in Figure 2. It consists of a shell, a damping pad, a transducer array, a matching layer, and an acoustical lens. The emissive power of each element is enhanced by using the acoustical lens. The transmitting and receiving array elements are controlled by the switch controller. The PMUT was characterized in water at the temperature 32 °C with a precision impedance analyzer (Agilent E4990A, Agilent Technologies, Santa Clara, CA, USA), and the results are shown in Figure 3. The PMUT’s resonant frequency is 3.5 MHz with an impedance of 67 Ω and a static capacitance of 665 pF. The values of impedance and capacitance provide a basis for the design of a 16 channels ultrasonic transmitting/receiving acquisition circuit. The frequency of 3.5 MHz of the ultrasound transducer has a broad application in the soft tissue medical imaging. PMUT’s array spacing is 1 mm, hence the 128 array elements can meet the breast imaging the dimension requirement of the 3D imaging.

**Figure 4:**
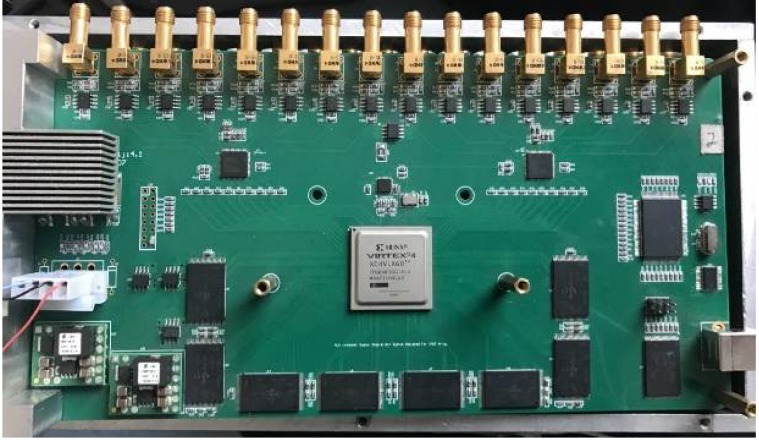
Ultrasonic transmitting/receiving acquisition circuit.

(b) Ultrasonic signal transmitting/receiving circuit:

Working conditions are determined based on the study of the ultrasonic transducer, the programmable 16 channels ultrasonic transmitting/receiving acquisition circuit is shown in Figure 4, which was designed by our research group. The data acquisition system is designed in the AD9222 core chip, with a sampling frequency of 12 MHz, a large capacity flash memory that is used as temporary storage medium, and a USB2.0 transmission bus. This system can be used in the 3D ultrasound imaging ring system to create the desired focus imaging technology. The advantage of this system is that it can achieve imaging performance far beyond a single channel [20].

Generally, the absorption coefficient of the soft tissue is 0.6~0.7 dB/(cm/MHz) [29]. With the increase of detection depth, the ultrasonic echo signal will be attenuated. Therefore, multi-channel is used to transmit and receive ultrasound signals to increase the detection depth and thereby producing high resolution images [30].

**Figure 5:**
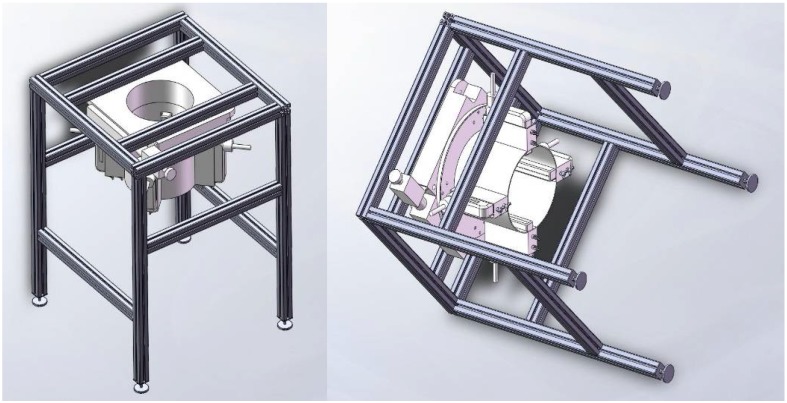
Experimental setup for the 3D ultrasound imaging ring system.

(c) Mechanical alignment device:

It is composed of an electric rotary table with a polymethyl methacrylate (PMMA) fixed device and a bracket made of stainless steel. Four 1 × 128 PMUT linear arrays were installed vertically in the PMMA fixed device, creating a 90° cross-symmetric structure as shown in Figure 5.The PMMA device is a cylindrical structure, which is controlled by an electric rotary table to realize circular scanning. The diameter of the PMMA device and the electric rotary table is equal. Commonly, the ultrasound transducer needs water as the coupling medium. Whereas, the electric rotary table that we used should be placed in air. Hence, the electric rotary table was installed inversely on the bracket. The prone examinee’s pendant breast is placed through the opening in the electric rotary table and into the water tank.

The operation of the system is as follows:

(1) The storage water tank is filled and the water is heated to a constant temperature by a heater. The temperature is maintained at 32 °C for patient comfort and to bring the water tank sound speed closer to physiologic levels.
(2) The prone patient’s pendant breast is placed through the electric rotary table opening into the water tank. Scanning is accomplished with the electric rotary table moving the transducer assembly to the PMMA fixed device, whereupon the transducer assembly begins continuously acquiring reflectivity ultrasound signals as the transducer rotates through 90°. A vertical slice is acquired using linear scanning method at a fixed angle and a horizontal slice is obtained through circular scanning method when 90° scan is completed at various angles with predetermined interval.
(3) Multiple slices are gained by shifting the PMUT array element until the entire breast or object is scanned. A 3D breast image can be reconstructed by merging multiple slices. Finally, it is visualized on a personal computer workstation.

## 3. Results

**Figure 6:**
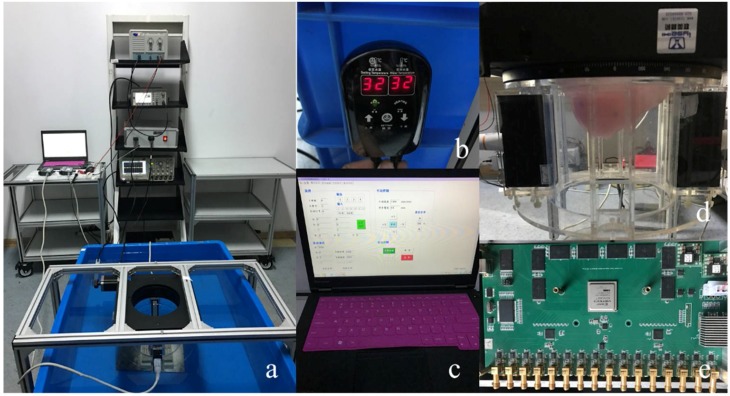
(**a**) Experimental platform system. Placement of breast in the ring; (**b**) Constant temperature heater; (**c**) Personal computer workstation; (**d**) Mechanical alignment device. Transducer ring configuration; (**e**) Ultrasonic signal transmitting and receiving circuits.

According to the novel 3D ultrasound imaging ring system shown in Figure 1, experimental platform system is constructed as shown in Figure 6.

### 3.1. Methods

**Figure 7:**
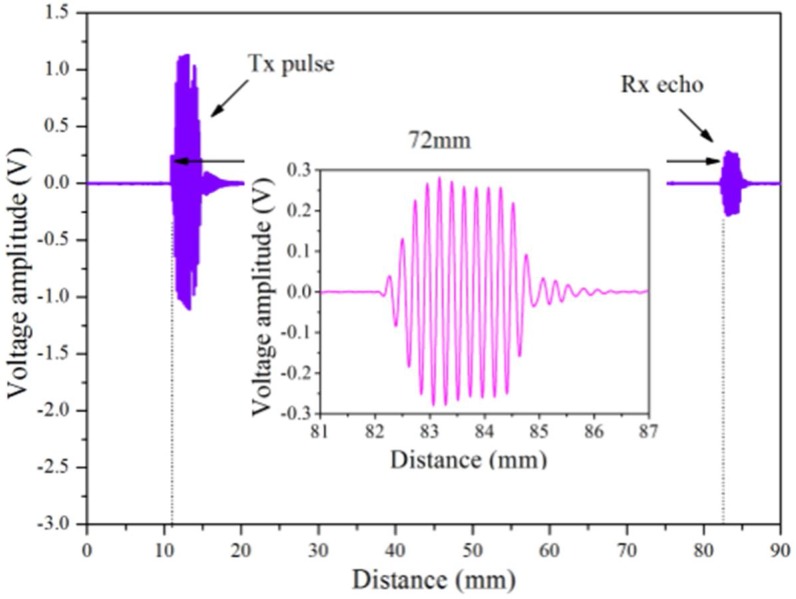
Ultrasound pulse echo signal with a zoomed in view of the echoed signal.

It is known that when an ultrasonic pulse propagates in an object or soft tissue, a reflection of this pulse will occur every time it passes an interface layer where the refractive index is different [3]. A tomography image reconstruction can be accomplished by using sound reflection, transmission, or a combination of reflection and transmission. Although this is an early stage study of the breast ultrasound tomography imaging technology, the classic reflection ultrasonic imaging technology is used to verify the feasibility and validity of this system. In this experiment, the four 1 × 128 PMUT linear arrays are used for not only transmitting, but also receiving ultrasound signals, respectively. The experiment was conducted in the water tank, at a temperature of 32 °C. The PMUT array was driven by a signal generator (Agilent 33521A) and amplified by a power amplifier (HA205) [21]. The N-th element is excited by an AC sine voltage that has a pulse repetition interval of 1ms, an operating frequency of 3.5 MHz and an amplitude of 100 Vpp. The pulse echo is received by the same N-th element. Both the ultrasound pulsed signal and echoed signal are shown in Figure 7.

The characteristic values are normalized to provide an intuitive view of the relative relationship of the transmitting and receiving signals. The speed of sound in water is 1540 m/s [12]. The distance is obtained by calculating the time difference between the transmitted signal and the received signal, times the speed of sound, and then divided by 2, as shown in Equation (1):

(1) $$s=\frac{t\times c}{2}$$

**Figure 8:**
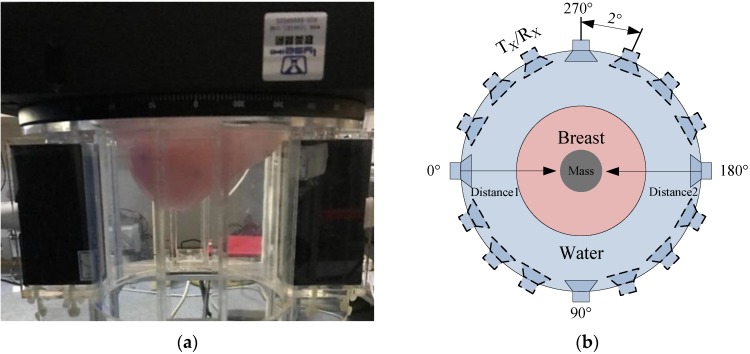

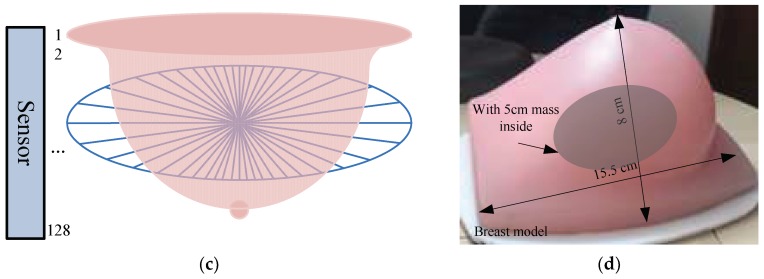
Ultrasound tomography transducer acquisition setup. (**a**) Placement of breast in the ring; (**b**) Transducer ring configuration platform; (**c**) Slice maps of ultrasound tomography imaging; (**d**) Breast Model.

The results indicate that the object distance from the transducer is 72 mm. As the different impedance of the target object is detected, the amplitude and distance of pulse echo signal will change, so the detection of the soft tissue can be accomplished by using the pulse echo method. In addition, the size and location of masses can be determined through subtracting distance 1 and distance 2 from the diameter (180 mm) of the electric rotary table via the echo pulse method, as shown in Figure 8b.

The setup of the ultrasound tomography transducer acquisition is shown in Figure 8. The breast model is placed through the electric rotary table opening into the water tank, which is demonstrated in Figure 8a. The transducer ring configuration platform is created as shown in Figure 8b. And the number of the probe can be adjusted. In the experiment, the four PMUT linear arrays are placed 90 degrees cross vertically which were set at 0 degrees (PMUT 1), 90 degrees (PMUT 2), 180 degrees (PMUT 3), and 270 degrees (PMUT 4), respectively. The distance of opposite PMUT’s is 180 mm. Set1(PMUT 1, 2) and Set2(PMUT 3, 4) work alternately. During the testing of the circular scanning imaging technology, and an image slice is obtained by rotating the transducer 90 degrees with an interval of 2 degrees with the electric rotary table. The more probes, the shorter the acquisition time of data. The precision of the electric rotary table is 0.05°, which allows for a theoretical achievement of 7200 echo pulse signals. 128 horizontal slices are achieved by rotating the N-th element through a full circle. A slice map of ultrasound tomography imaging is shown in Figure 8c. It is the basis of 3D breast imaging.

According to the characteristics of breast detection, the circular scanning method was used. A stereo scan of the breast model can be completed when the probe is rotated 90 degrees. The breast model is shown in Figure 8d. The processing step of a horizontal slice data is as follows: (1) store data in columns which each column is an ultrasonic echo data; (2) a Butterworth filter process; (3) envelope detection; (4) logarithmic compression; (5) coordinate transformation using the center of the electric rotate table as a reference point; (6) morphological processing including a bicubic filling algorithm and an inflation algorithm. Then, a horizontal slice image can be produced. Most importantly, the algorithm only needs to detect the half depth of the detection target, which can realize full depth imaging and reduce the power consumption of the ultrasonic transducer.

### 3.2. Experiments and Results

#### 3.2.1. Iron Model Imaging

**Figure 9:**
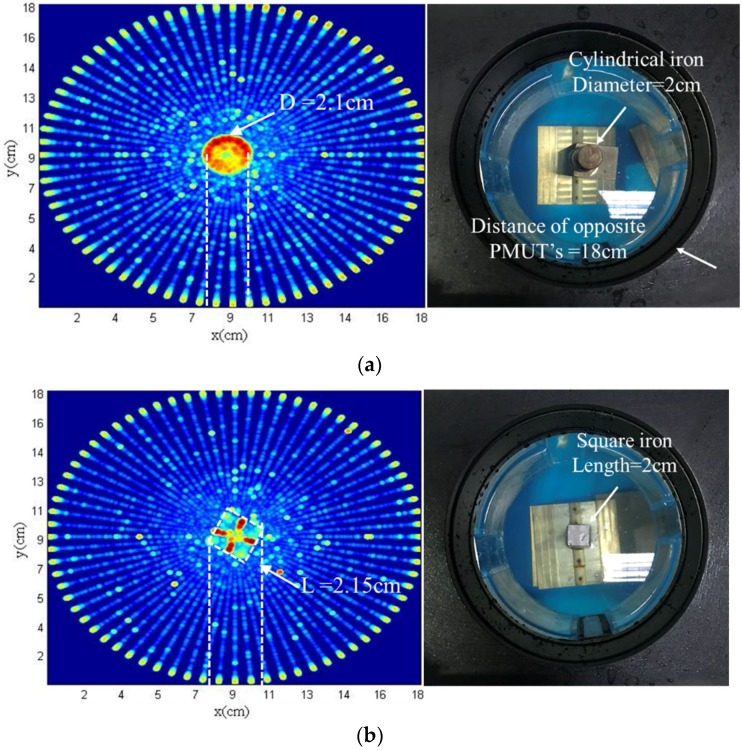
Ultrasonic tomography of iron using the ring system. (**a**) Cylindrical iron imaging and configuration; (**b**) Square iron imaging and configuration.

In this section, ultrasound tomography imaging of an iron model in different shapes were obtained by circular scanning using the ring system. A cylindrical iron and a square iron are experimented using the ring system as shown in Figure 9. The operation process of the PMUT has been described previously, and thus will not be described in detail here. The ultrasonic tomography images of the cylindrical and square iron models are shown in Figure 9a,b, respectively.

Through analysis of the relative position and distance of the scanned model, the size and the outline of the iron model can be delineated. The diameter of the cylindrical iron is 2.1 cm measured by the experiment. The relative error is 5%. Whereas, the length of the square iron is 2.15 cm measured by the experiment. The relative error is 7.5%. Measured values are in conformity with the actual values. However, the ultrasonic detection of the cylindrical iron is better than the square iron model, mainly because sound wave reflection from a cylindrical surface is easier to detect due to smaller angles of incidence, and thus the greater amplitude of reflected wave. The detection of contour is related to the change of incident angle. The smaller the change of incident angle, the clearer the contour. The preliminary imaging was implemented, which verified the feasibility of the circular scanning imaging system. 

#### 3.2.2. Breast Model Imaging

The imaging of iron was used of initial testing of the system. The setup was evaluated with a breast model (15.5 cm × 8 cm) with a 5 cm mass to verify the applicability of the system. Three groups of experiment were designed by changing the angle of rotation while keeping the PMUT array element constant.

**Figure 10:**
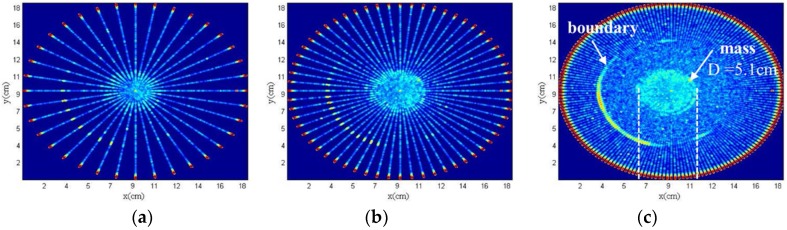
Ultrasonic tomography of breast model using the ring system with an (**a**) angle of 10°; (**b**) angle of 6°; (**c**) angle of 2°.

The angle is determined by the electric rotary table, the precision of electric rotary table is 0.05°, which allows for the theoretical acquisition of 7200 echo pulse signals each full circle. The ultrasonic image of breast model using ring system at intervals of 10°, 6° and 2° are shown in Figure 10. The preliminary imaging is accomplished, the size of the mass is about 5.1 cm in the center of the breast model. The boundary of the breast model also can be distinguished. The results show that the test value of the mass is consistent with the actual value, and the setup can detect two substances. The images acquired from the different intervals demonstrate the relationship between scanning interval and image quality. Additionally, the resolution of the imaging improves with the decreasing interval angle.

**Figure 11:**
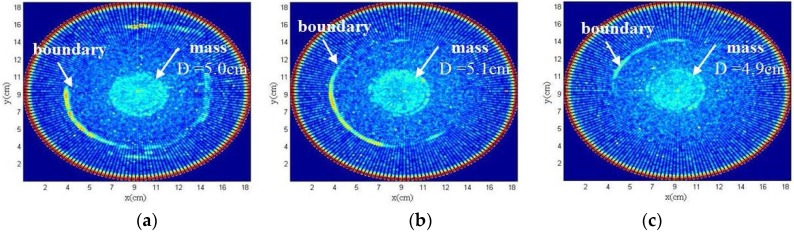
Ultrasonic tomography of breast model using the ring system. (**a**) Slice *N* = 30; (**b**) Slice *N* = 50; (**c**) Slice *N* = 70.

By using the 1 × 128 PMUT linear array, 128 horizontal slices are produced from element 1 through 128 at intervals of 2°. The reconstruction time per slice is 534.4 s by using HP workstation (16 G). The element spacing is 1 mm. Elements 30, 50, and 70 are shown in Figure 11, in which the mass can be seen in Figure 11a–c. The location of the masses is in the center of the breast model. The size of the masses is about 5.0 cm, 5.1 cm, 4.9 cm respectively. Furthermore, the mass was clearly detected. However, the size of the mass is diverse due to transition to different slices of the breast model. Characteristic information of soft tissue each slice is different. Therefore, the setup can distinguish objects from the background even inner structures. The breast model used in this experiment is shown in Figure 8d. The boundary of the breast model also can be distinguished. However, the boundary is not complete. This is due to the uneven shape of the breast model. The tilted side is not easily detected because the incident angle is relatively large. Besides, only one element was used to emitting and receiving at the same time. If we use focus technology, the quality of image will be improved. Experimental results verified the effectiveness of this ring system, in which 3D breast imaging will be accomplished through multilayer slice data reconstruction.

## 4. Discussion and Conclusions

In this project, a novel 3D ring imaging system with a rotatable 1 × 128 PMUT linear array is achieved. Based on the characteristics of breast detection, a circular scanning method is proposed. According to the characteristics of circular scanning, the corresponding ultrasound tomography is designed. The algorithm only needs to detect the half depth of the detection target, which can realize full depth imaging and reduce the power consumption of the ultrasonic transducer. Through the experimental analysis of ultrasonic imaging of a cylindrical iron, a square iron and a breast model, the size, location, and outline of a hypothetical mass can be distinguished from soft tissue. This setup is mainly for the detection of breast lesion, through the electric rotary table control, combined with ultrasonic transducer is placed vertically (adjustable number), a circular scanning (adjustable interval angle) can realize 3D scanning of mammary gland. By using rotatable line array instead of a cylindrical array, it can decrease the difficulties of sensor’s fabrication processing and overcome the poor consistency and poor reliability of a cylindrical array. Compared with the existing ultrasonic equipment, this setup has a simple structure and low cost. Moreover, the setup can obtain both reflected signals and transmission signals. Multi-modal imaging studies can be carried out. However, the 3D scanning relies on mechanical rotation, thus causing inevitable water disturbance and more data acquisition time. Test results are consistent with the theoretical values. The ultrasound tomography imaging validates feasibility of the system and validity of the algorithm, with additional advantage of portability.

## 5. Patents

Chinese Patent: The breast ultrasound imaging system and the testing method based on CMUT ring array (Application No. 2017105534314).
